# Human orf virus (family *Poxviridae*) infection following a lamb bite in Hungary

**DOI:** 10.1007/s00705-024-06002-w

**Published:** 2024-03-02

**Authors:** Csongor Németh, Ákos Boros, Endre Mészáros, Csaba Gyömörei, Ervin Albert, Péter Pankovics, Gábor Reuter

**Affiliations:** 1 Department of Dermatology, Venereology and Oncodermatology, Medical School, University of Pécs, Pécs, Hungary; 2 Department of Medical Microbiology and Immunology, Medical School, University of Pécs, Szigeti út 12, H-7624 Pécs, Hungary; 3 Szent Rókus Hospital, Baja, Hungary; 4 Department of Pathology, Medical School, University of Pécs, Pécs, Hungary; 5 Department of Pathology, University of Veterinary Medicine, Budapest, Hungary

## Abstract

Human orf disease (called ecthyma contagiosum or contagious/infectious pustular dermatitis in animals) was confirmed on the fingers of both hands of a 24-year-old female, after feeding diseased lambs with a nursing bottle in April 2023. In addition to skin symptoms, she had low-grade fever (37.6°C) and swollen lymph nodes in both axilla. The presence of orf virus (genus *Parapoxvirus*, family *Poxviridae*) was confirmed, and this strain, Baja/2023/HUN (OR372161-OR372163), was found to have > 98% nucleotide sequence identity to sheep-origin orf viruses in four tested genome regions (ORF011/B2L, ORF019, ORF020/VIR, and ORF056). This is the first report of a human case of infection with the neglected zoonotic orf virus in Hungary.

**Supplementary Information:**

The online version contains supplementary material available at 10.1007/s00705-024-06002-w.

Orf disease in humans, also known as ecthyma contagiosum or contagious/infectious pustular dermatitis when it occurs in animals, is a zoonotic infection associated with dermatotropic parapoxvirus (genus *Parapoxvirus*, family *Poxviridae*) [1https://ictv.global/report/poxviridae]. Orf virus primarily infects sheep and goats, most commonly juvenile animals, and is transmitted to humans through direct or indirect contact with an infected animal or fomite. In ungulates, it causes vesiculo-ulcerative pustular lesions that are localized to the skin and the mucosal surfaces of the oral cavity (called “sore mouth” or “scabby mouth”) and the nasal cavity. Human orf virus infection is manifest 3–15 days after inoculation on the exposed part of the skin, most commonly on the dorsal aspect of the hands and fingers. The skin symptoms undergo a characteristic morphological change, with the formation of papules, nodules, vesicles, blisters, pustules, erosions, and ulcers over time [2]. The disease is usually self-limiting in immunocompetent individuals, resolving spontaneously within 3 to 8 weeks [3]. Reinfection is possible [3], but no specific treatment is needed.

Orf virus infections and the resulting diseases seem to be neglected in Europe [4]. There is a lack of systematic statistical data about the current prevalence and seroepidemiology of orf virus in livestock and the endemic and epidemic status of the virus in European countries. Descriptions of a few individual sporadic human cases [2, 5–9] has drawn attention to the fact that orf virus infection is probably underdiagnosed in both animals and humans.

Here, we report the first confirmed human case of orf virus infection and disease in Hungary associated with a lamb bite during animal feeding.

Viral DNA was isolated from a skin scraping specimen using a High Pure Viral Nucleic Acid Kit (Roche) according to the manufacturer’s instructions. Four regions of the orf virus genome were amplified by PCR using sense (F) and antisense (R) primer pairs designed to correspond to the ORF011/B2L (F: 5′-TCCCTGAAGCCCTATTATTTTTGTG-3′/R: 5′-GCTTGCGGGCGTTCGGACCTTC-3′), ORF019 (F: 5′-CGGTCTCTGAGGTAGTCCCT-3′/R: 5′-TCACCTGCACCATCACGATAT-3′), ORF020/VIR (F: 5′-AAAATTAGAAGCTGATGCCGCAG-3′/R: 5′-CCACAATGGCCTGCGAGTG-3′), and ORF56/RNA polymerase (F: 5′-CATCCCCAAGGAGACCAACGAG-3′/R: 5′-TCCTCGTCGCCGTCGAAGTC-3′) regions. Primers for the ORF56 region are suitable for amplification of poxviruses of the genera *Molluscipoxvirus* and *Parapoxvirus* [10]. PCR products were sequenced directly and run on an automated sequencer (3500 Genetic Analyzer, Applied Biosystems).

A 24-year-old female, working with sheep as a part-time job in Bács-Kiskun County, Hungary, was referred to our university dermatological ambulance on May 8, 2023, with ulcerated, burning-itching papules on her fingers in both hands (Fig. 1E–G). Her symptoms had started ~ 2 weeks earlier (Fig. 1A–D) after feeding lambs with a nursing bottle without wearing personal protective equipment. One of the animals bit the fifth finger of her left hand during feeding. She reported that several lambs in the herd had sores on their mouths, which she had treated using antiseptic lotions (povidone-iodine and alcoholic solution). She had low-grade fever (37.6°C) 4 days before her initial medical examination, as well as swollen (reactive) lymph nodes (max. 3.2 cm in length) in the right and left axilla, which was confirmed by ultrasonography. Earlier, the patient had visited the local family doctor (on May 3) and the local infectious diseases and dermatology wards (on May 4) in her city of residence, where serological test results for *Bartonella henselae* (cat scratch disease), *Bartonella quintana*, and *Francisella tularensis* (tularaemia) were negative. No pathogenic bacteria or fungi were cultured from the wound exudate. Blood leukocyte count, C-reactive protein (CRP = 1.0 mg/L), and erythrocyte sedimentation rate (ESR = 3mm/h) were in the normal range, but a mild monocytosis (1.02 g/L, 16%) could be detected. After wound excision, oral azithromycin treatment (500 mg on the first day, then 250 mg for 4 days) was prescribed; however, her symptoms did not improve.

**Fig. 1:**
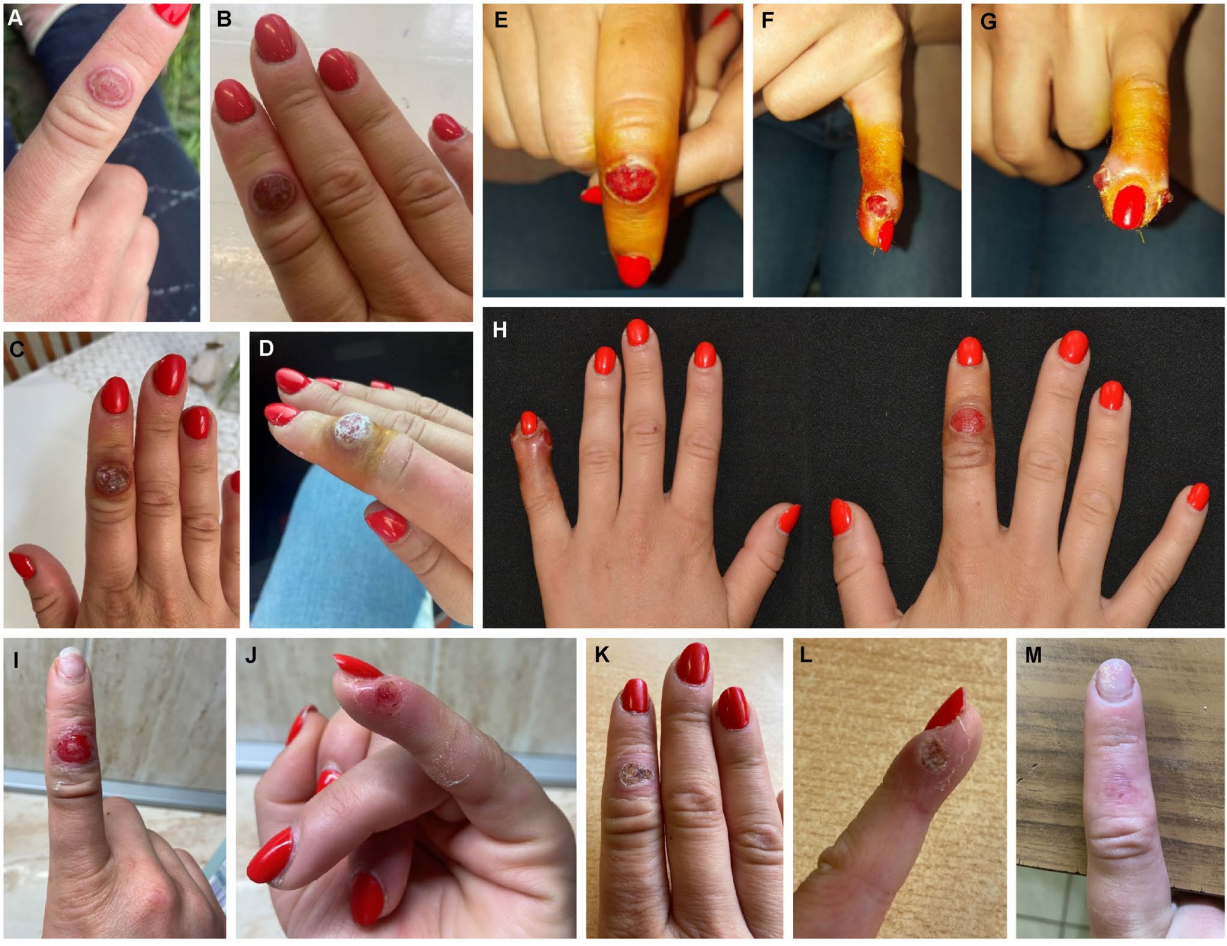
Characteristics and progression of the skin lesions over time on both sides of the left little finger (fifth, bitten finger) and the right index (second) finger. At first, a red macule or papule appeared (no image), which then became larger, and a targetoid appearance developed, with a paler central halo and a red outer ring with a diameter of 1.5 cm (**A**–**H**, photos from before May 7). The raised centre of the lesion appeared to fluctuate (**A**). In the next stage, the centre eroded (**E**–**G**, photo from May 8; **I**, **J**, photo from May 13), resulting in a seeping crusted surface (**K**, **L**, photo from May 23). Under the crust, the lesions healed without scarring within 8 weeks (**M**, photo from June 25). The photographs were taken by the patient (and used with the patient’s permission)

On physical examination on May 8 (Fig. 1E–G), there were two 6-mm eroded symmetric papules on both sides of the left little finger (the fifth, bitten finger, Fig. 1F-G) and a 10-mm eroded papule on the right index (second) finger (Fig. 1E). There was no sensation or circulation disturbance in the affected fingers. X-ray examination was negative. Based on the clinical picture and the patient’s medical history, the possibility of orf virus infection was suspected. An allergic reaction with mild papular rash appeared on the back of the hands and on the left wrist on May 11 (Supplementary Fig. S1).

Skin scraping specimens collected on May 10 (Fig. 1H) all tested positive for orf virus DNA by the PCR method followed by Sanger sequencing using primer pairs designed to amplify four different genome regions: open reading frame ORF056 (RNA polymerase I subunit A), ORF011 (major envelope gene, B2L), ORF019, and ORF020 (viral interferon resistance gene, VIR), with the last two of these producing overlapping PCR amplicons. The ORF011 region of the strain from our patient (Baja/2023/HUN, GenBank accession no. OR372161) had 99.2% nucleotide sequence identity to the corresponding sequence of the sheep-origin orf virus strain TVL/USA (MN454854), and the ORF056 and ORF019/ORF020 regions (OR372162-3) were 98.74% and 99.67% identical, respectively, to those of strain NZ2/New Zealand (DQ184476) [11], as the closest matches using GenBank BLASTn. Phylogenetic analysis based on the ORF011 and ORF020 genes showed that strain Baja/2023/HUN clustered together with sheep-origin orf viruses (Fig. 2).

**Fig. 2:**
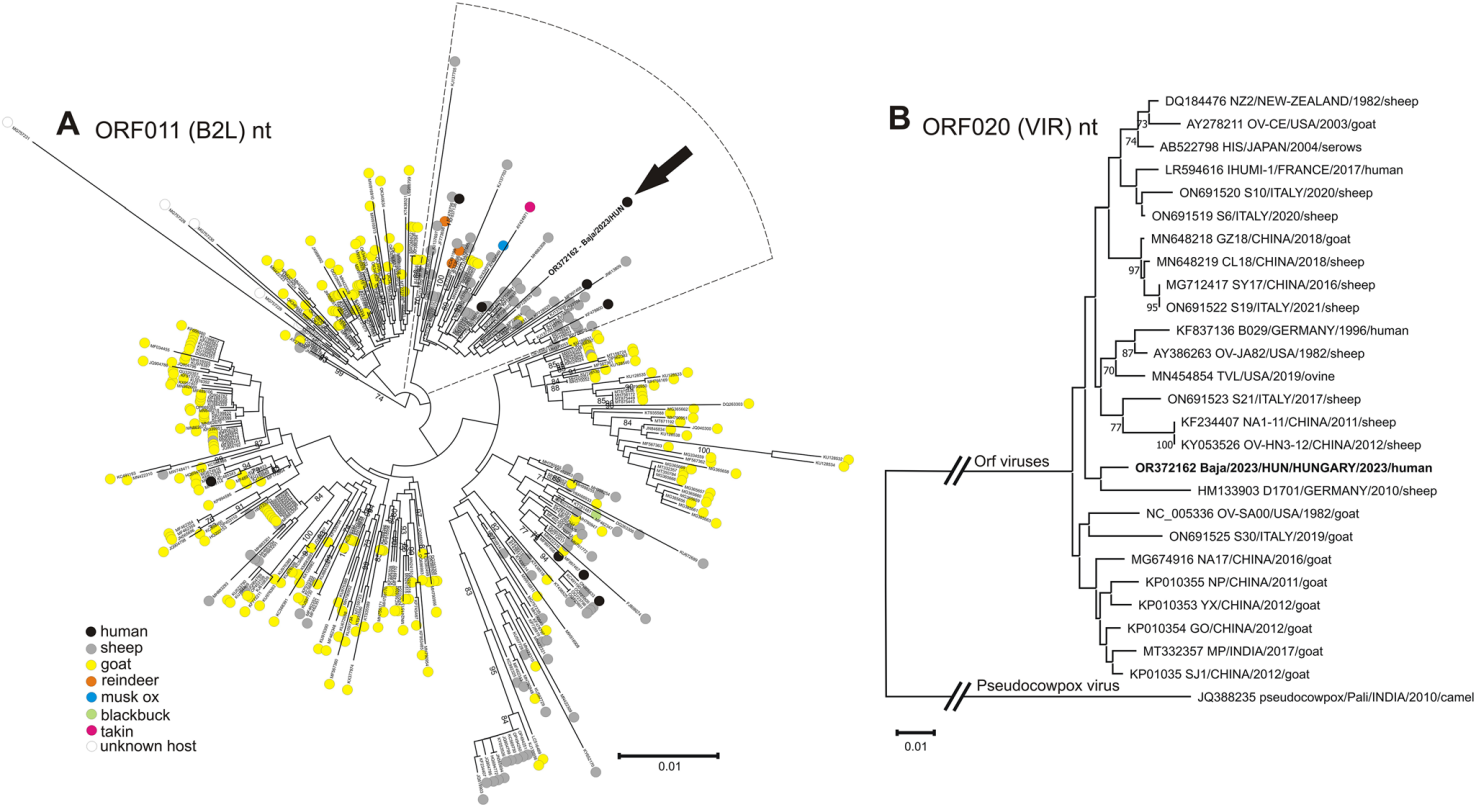
Phylogenetic analysis of orf viruses (genus *Parapoxvirus*) based on the nucleotide sequences of (**A**) ORF011 (B2L) and (**B**) ORF020 (VIR). The study strain Baja/2023/HUN (OR372161-OR372163) is indicated by bold letters (and a black arrow in panel A). In panel A, a total of 386 orf virus sequences were collected from the GenBank database and analysed. The host species origin of the orf viruses is indicated by colour coding (circle). The strain from this study (Baja/2023/HUN) clustered together with sheep-origin (gray circle) orf viruses, which formed a distinct clade (cake slice with dotted line). In panel B, orf virus strains with complete genome sequences available in the GenBank database [17] and those whose sequences were most similar to that of the study strain were analysed. The sequences are indicated by accession number, strain name, country of origin, presumptive year of identification, and host species name. Both trees were generated using MEGA software version 11 [18] by the neighbor-joining method with the Jukes-Cantor model and 1000 bootstrap replicates. Only bootstrap values higher than 70 are indicated in the trees. The tree in panel A was visualized using the iTOL web server (https://itol.embl.de/)

A punch biopsy of the skin was performed on May 17 and stained with hematoxylin-eosin (Fig. 3). The biopsy material showed signs of skin regeneration (Fig. 3). Virus particles with a morphology characteristic of orf virus could not be detected in the epidermis by transmission electron microscopy (data not shown).

**Fig. 3:**
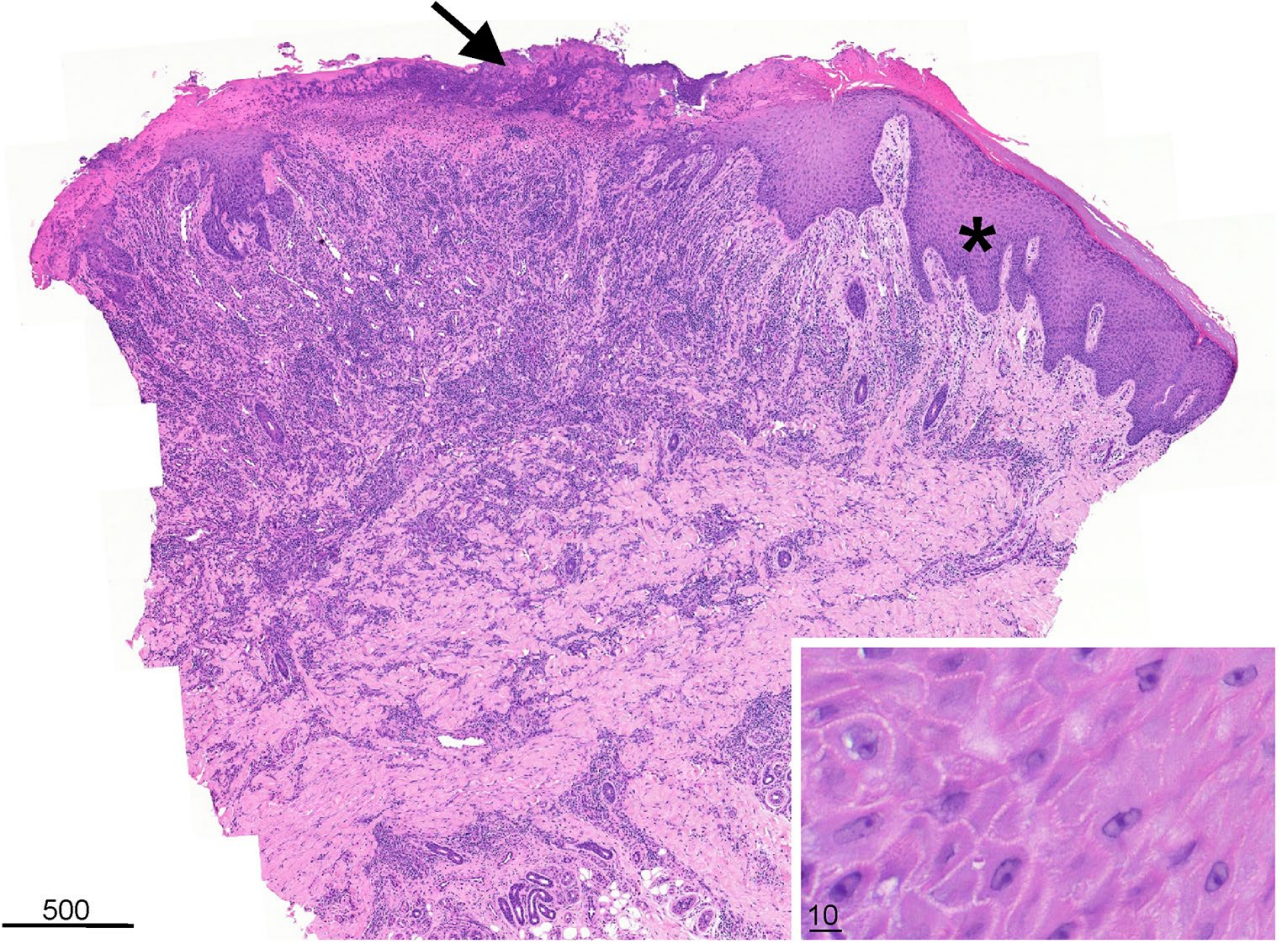
Scanning view (hematoxylin–eosin staining, 3x magnification). A skin biopsy (collected on May 17) from the edge of a lesion was stained with hematoxylin and eosin, showing an ulcerated surface with overlying crust (see the top of the histological section, black arrow). The non-ulcerated epidermis shows irregular acanthosis and hypergranulosis (black asterisk). There is a dense chronic inflammatory infiltrate in the upper and deep dermis. Inset: High-magnification view (hematoxylin–eosin staining, 100x magnification) of the spinous layer. No intracytoplasmic inclusion bodies were observed. The units of the scale bars are micrometers

The patient’s treatment consisted of disinfecting the affected fingers and covering the wounds with sterile bandages. In the follow-up 6 weeks after the contact, her lesions showed improvement (Fig. 1I–L) and healed nearly completely (Fig. 1M). No further primary or secondary human orf cases were reported related to this event.

Orf virus infection is an occupational hazard, especially among shepherds, butchers, farmers, wool shearers, and veterinarians, but it also occurs in nonoccupational settings (among zoo visitors, hobby farmers, animal slaughtering during religious sacrifices, etc.) [3]. Human-to-human transmission has been documented in some cases [12], and autoinoculation is thought to be rare. In many countries, human orf is not a notifiable disease, and there are no international data collections indicating the actual number of infections. The majority of the diseased individuals probably do not seek medical attention, as animal keepers are familiar with the mild and self-limiting course of the disease, and they may fear stigmatisation and possible economic consequences if the infection is reported. Furthermore, many health care professionals do not think about this type of infection, and cases are often misdiagnosed. In Hungary, the frequency of orf virus infection in animals has not been reported in the past 70 years [13], and human cases are not registered at all. This is therefore the first confirmed – and documented – case of human orf virus infection and disease in the country.

Within the framework of clinical microbiological diagnostics, the conventional PCR method is sufficient to confirm the clinical diagnosis of orf virus infection. However the determination of the complete genome sequence of the virus can provide additional evolutionary, chrono-phylogeographical, and host species information. Phylogenomics could improve our understanding of the evolutionary divergence of orf viruses from sheep from those from goats. Based on the results of the sequencing and phylogenetic analysis, which is traditionally performed on two regions (ORF011/B2L and ORF020/VIR) of the orf virus genome [4, 9, 10], the human strain Baja/2023/HUN showed the closest relationship to orf viruses originating from sheep. An attempt to obtain test samples from the affected and diseased sheep through the animal health authority was not successful. Although there is no direct microbiological evidence, the known contact with animals with orf-like symptoms (“scabby mouth”) is likely to have been the primary source of the infection. Human orf disease is confirmed based on the patient’s medical history, clinical symptoms (typical dermatological findings) [14, 15], and microbiological (PCR and sequencing) and pathological (histology) laboratory methods. Other skin diseases that are considered in differential diagnosis include herpetic whitlow, milker’s nodules, cowpox, Mpox (monkeypox), cutaneous anthrax, tularemia, cutaneous leishmaniasis, infection of *Mycobacterium marinum*, deep fungal infections, pyogenic granulomas, keratoacanthoma, and malignant tumours, among others [16].

Although orf is a self-limiting and often neglected disease, orf virus should be handled as a potential re-emerging pathogen – especially in the light of the recent sexually transmitted Mpox pandemic – in both animal and human health.

### Electronic supplementary material

Below is the link to the electronic supplementary material.

**Supplementary Fig. S1** An allergic reaction with mild papular rash appearing on the backs of both hands (A and B, photos from May 11; used with the patient?s permission) and the left wrist (not shown)
