# Supplementary figures and images for: Human orf virus (family Poxviridae) infection following a lamb bite in Hungary
Source: Arch Virol. 2024 Mar 2;169(3):59. doi: 10.1007/s00705-024-06002-w (PMC10908620; doi:10.1007/s00705-024-06002-w)

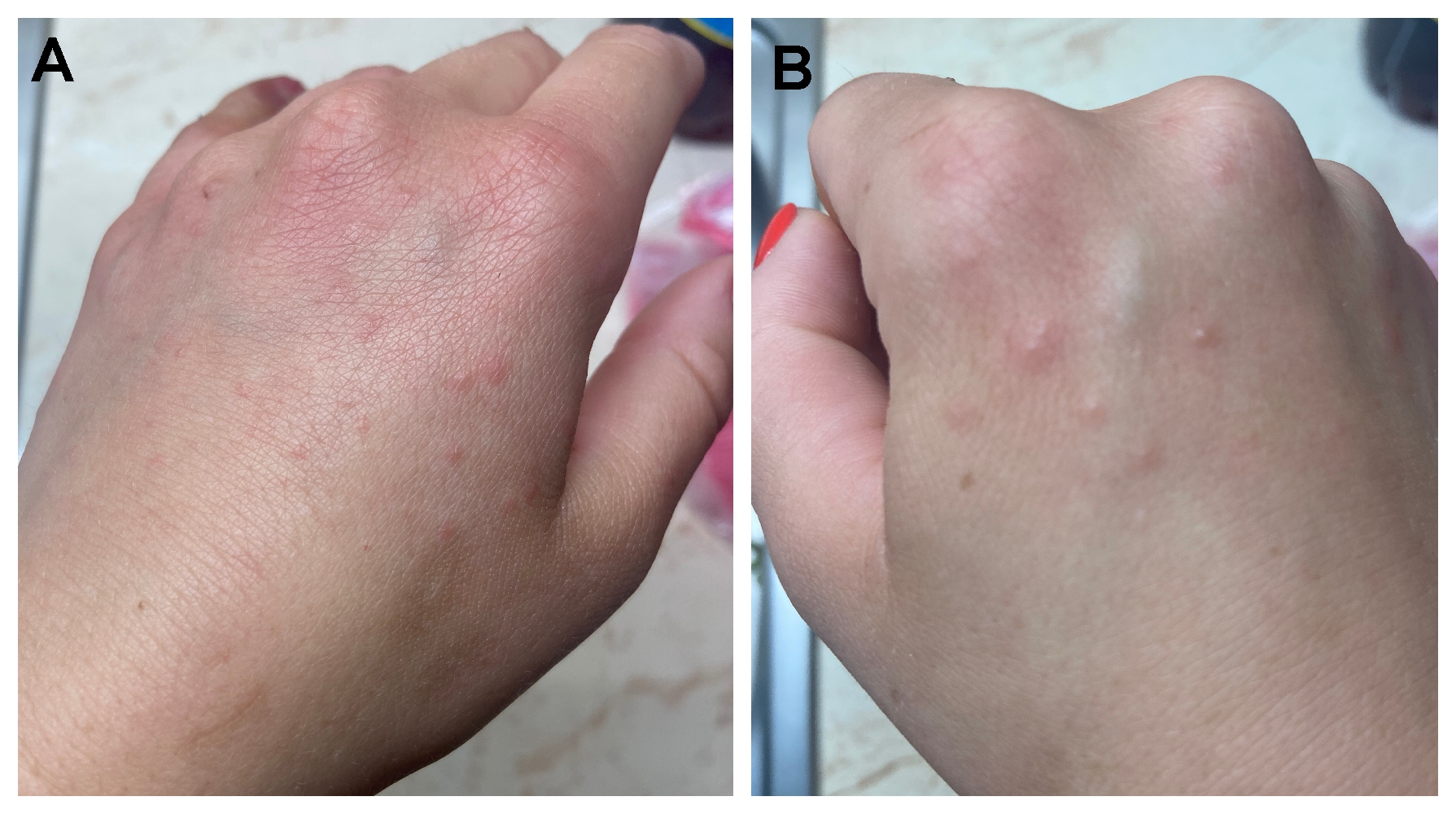

Supplement: Supplementary file 1 — Supplementary Fig. S1 An allergic reaction with mild papular rash appearing on the backs of both hands (A and B, photos from May 11; used with the patient?s permission) and the left wrist (not shown) [file 705_2024_6002_MOESM1_ESM.jpg]
